# *CARMAL* Is a Long Non-coding RNA Locus That Regulates *MFGE8* Expression

**DOI:** 10.3389/fgene.2020.00631

**Published:** 2020-06-17

**Authors:** Sébastien Soubeyrand, Majid Nikpay, Paulina Lau, Adam Turner, Huy-Dung Hoang, Tommy Alain, Ruth McPherson

**Affiliations:** ^1^Atherogenomics Laboratory, University of Ottawa Heart Institute, Ottawa, ON, Canada; ^2^Ruddy Canadian Cardiovascular Genetics Centre, University of Ottawa Heart Institute, Ottawa, ON, Canada; ^3^Center for Public Health Genomics, University of Virginia, Charlottesville, VA, United States; ^4^Children Hospital of Eastern Ontario Research Institute, Ottawa, ON, Canada; ^5^Department of Biochemistry, Microbiology and Immunology, University of Ottawa, Ottawa, ON, Canada; ^6^Department of Medicine, University of Ottawa Heart Institute, Ottawa, ON, Canada

**Keywords:** lncRNA, *CARMAL*, *RP11-326A19.4*, *MFGE8*, gene expression, transcription, *SBDS*

## Abstract

Genome-wide association studies have identified several genetic loci linked to coronary artery disease (CAD) most of them located in non-protein coding regions of the genome. One such locus is the CAD Associated Region between *MFGE8* and *ABHD2* (CARMA), a ∼18 kb haplotype that was recently shown to regulate vicinal protein coding genes. Here, we further investigate the region by examining a long non-coding RNA gene locus (*CARMAL/*RP11-326A19.4/AC013565) abutting the CARMA region. Expression-genotype correlation analyses of public databases indicate that *CARMAL* levels are influenced by CAD associated variants suggesting that it might have cardioprotective functions. We found *CARMAL* to be stably expressed at relatively low levels and enriched in the cytosol. *CARMAL* function was investigated by several gene targeting approaches in HEK293T: inactive CRISPR fusion proteins, antisense, overexpression and inactivation by CRISPR-mediated knock-out. Modest increases in *CARMAL* (3–4×) obtained via CRISPRa using distinct single-guided RNAs did not result in consistent transcriptome effects. By contrast, *CARMAL* deletion or reduced *CARMAL* expression via CRISPRi increased *MFGE8* levels, suggesting that *CARMAL* is contributing to reduce *MFGE8* expression under basal conditions. While future investigations are required to clarify the mechanism(s) by which *CARMAL* acts on *MFGE8*, integrative bioinformatic analyses of the transcriptome of *CARMAL* deleted cells suggest that this locus may also be involved in leucine metabolism, splicing, transcriptional regulation and Shwachman-Bodian-Diamond syndrome protein function.

## Introduction

Genome-Wide Association Studies (GWAS) have identified genomic variants that statistically partition with numerous diseases. With regard to cardiovascular disease (CAD) more than 160 loci tagged by distinct single nucleotide polymorphisms (SNPs) distributed throughout the genome have been shown to associate with CAD risk (*p* < 10^–8^). Since the vast majority of these SNPs are situated in regions distant from genes, understanding of their role in disease processes will necessitate extensive follow-up mechanistic inquiries. Recently, we examined the role of a specific CAD associated 18 kb region in HuH-7 hepatoma cells and showed that it could regulate 2 distinct genes situated ∼150 kb away (*MFGE8* and *HAPLN3*) (Soubeyrand et al., 2019b). Both proteins are secreted and involved in cell adhesion. *MFGE8 (milk fat globule-EGFP factor 8)* encodes for lactadherin, which has been implicated in several physiological and cellular processes (Raymond et al., 2009). The physiological role of *HAPLN3* (hyaluronan and proteoglycan link protein 3) is less clear, although it has been linked to height (UK Biobank data) and cancer (Møller et al., 2017).

Our previous study focused on validated protein coding genes in *cis*, but in the course of the study the presence of a long non-coding RNA (lncRNA) gene, RP11-326A19.4, in close proximity to the CAD associated region was noted. Long non-coding RNAs are relatively long transcripts that resemble traditional mRNA but lack protein coding potential (Ransohoff et al., 2017). Recent estimates suggest that they outnumber protein coding genes and are expressed pervasively throughout the genome. Compared to protein coding genes they tend to be less conserved, more spliced, expressed at lower levels and enriched in the nucleus (Derrien et al., 2012).

A current controversy is the relative importance of the transcription product *per se* versus the underlying chromatin remodeling giving rise to the nascent transcript. This contentious issue stems in part from the low expression and conservation of lncRNA sequences, which beyond complicating functional inquiries, have been interpreted to signify a lack of transcript function. As the function of lncRNAs is presumed to require folding, conservation of structure and function may be retained despite low nucleotide conservation (Diederichs, 2014). An analysis of a subset of lncRNA loci suggests that in most cases, *cis* effects were mediated by chromatin remodeling itself (Engreitz et al., 2016). For some lncRNA loci however, considerable evidence support a role for the transcript itself (e.g., Xist, MALAT1, NEAT1) (Böhmdorfer and Wierzbicki, 2015). Finally, some lncRNAs operate outside the nucleus; indeed lncRNAs originating from the nucleus have been located in the cytosol and mitochondria (Noh et al., 2018).

Preliminary attempts to characterize the function of this lncRNA revealed a putative regulatory role in *IL6* expression, which we ultimately showed to be the result of an off-target effect by the CRISPRa system through intermediates that remain to be clarified (Soubeyrand et al., 2019a). Here we have characterized the *RP11-326A19.4* transcript in an effort to uncover its biological role. Mining publicly available data and presenting novel functional data, we provide evidence that this lncRNA, *CARMAL* (CAD Associated Region between *MFGE8* and *ABHD2* LncRNA), is expressed in the vasculature, indicating that the locus is active and potentially functionally important. Functional investigations point to both genome-wide and local roles, via the repression of *MFGE8* expression through mechanisms that remain to be clarified.

## Results

### Identification of a Long Non-coding RNA Enriched in Arterial Tissues

We recently characterized a “gene desert” region linked to CAD through a combination of fine-mapping approaches and functional assays (Soubeyrand et al., 2019b). The investigation revealed that the region could regulate various genes in *cis*, primarily *MFGE8*, a gene located 130 kb away, as a likely CAD relevant downstream mediator. While the analysis focused on well characterized genes, we also noted the presence of an annotated but otherwise uncharacterized gene classified as a long non-coding RNA (lncRNA) immediately abutting the region (Supplementary Figure S1). This putative lncRNA (*RP11-326A19.4/AC013565.1*) is expressed in a haplotype dependent manner, with the protective CAD allele being associated with reduced expression in the vasculature (Supplementary Figure S2). This last feature hinted at a possible vascular role, an interpretation further substantiated by its enriched expression in the vascular bed relative to other tissues (Supplementary Figure S3). To reflect the physical integration of this lncRNA within the CAD associated region between *ABHD2* and *MFGE8* (CARMA) gene hub, the lncRNA is denoted here as *CARMAL* (CARMA Associated lncRNA).

### *CARMAL* Is Stably Expressed at Low Levels in HEK293T and Coronary Models

**FIGURE 1:**
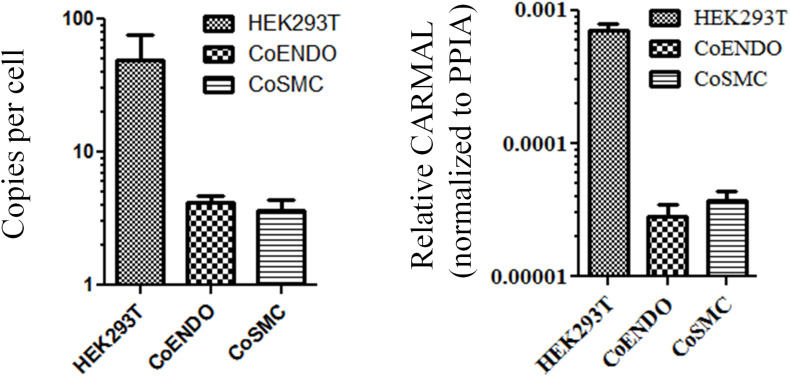
*CARMAL* is expressed at low levels. Copy number of *CARMAL* per cell **(left)** was estimated by droplet PCR while expression relative to *PPIA* **(right)** was determined by qRT-PCR. Data represent the mean of three biological replicates (±SD). CoENDO and coSMC refer to coronary endothelial cells and coronary smooth muscle cells, respectively.

Biological role is to a large extent tributary of expression level. For instance, enzymes which can undergo multiple rounds of catalysis (e.g., MAPK3) are considerably less abundant than structural proteins (e.g., TUBB) (Wang et al., 2015). Similarly more plentiful lncRNA are predicted to operate as structural scaffolds (e.g., *NEAT1*) or miRNA sponges (e.g., *DANCR*) whereas lower abundance RNA may point to more local roles (e.g., *ANRIL* and transcription regulation in *cis*). An early validation of the *RP11-326A19.4* transcript, described previously, confirmed its presence in HEK293T but its abundance was not investigated (Soubeyrand et al., 2019a). Expression levels of *CARMAL* were measured by droplet digital RT-PCR (ddPCR) in HEK293T and two primary coronary models using a pair of oligonucleotides targeting exon 1 and 2, shared by all reported splice variants of *CARMAL*. Expression was generally low, ranging from ∼50 copies per cell in HEK293T to only a few copies in the vascular models; consistent (relative) values were obtained by qRT-PCR after normalization to the housekeeper gene Peptidyl-prolyl cis-trans isomerase A (*PPIA*) (Figure 1).

### *CARMAL* Is Enriched in the Cytoplasm and Is Polyadenylated

**FIGURE 2:**
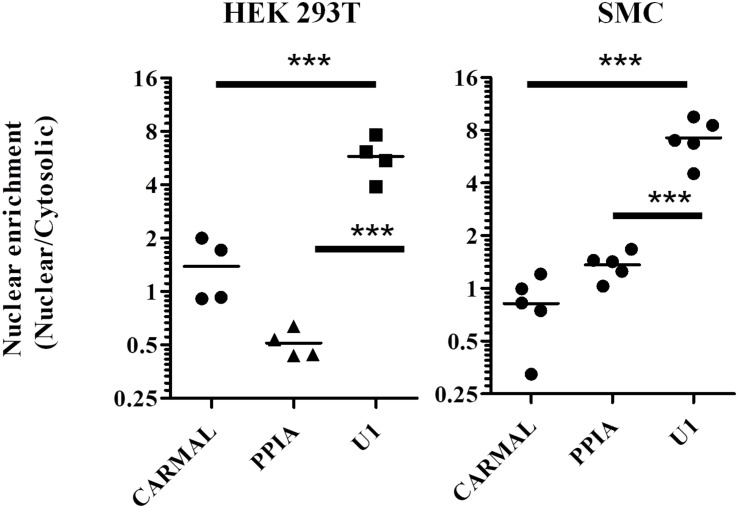
*PPIA* and *CARMAL* display similar cell fractionation profiles. Relative transcript enrichment in fractionated HEK293T and arterial smooth muscle (SMC) cells. Cells were fractionated sequentially into cytosolic and nuclear fractions, and the indicated RNAs within were quantified by qRT-PCR. Nuclear enrichment is defined as the ratio of nuclear to cytosolic signals. Individual data points represent independent fractionations. Statistically significant differences were tested using one-way ANOVA and Tukey’s *post hoc* test. *** indicates *p* < 0.005.

As biological function is contingent on localization, we next determined *CARMAL* subcellular distribution. Fractionation assays were performed on HEK293T cells as well as primary smooth muscle cells. In both cell types *CARMAL* fractionated similarly to *PPIA*, a mature (cytosolic) mRNA, in showing a pattern distinct from U1, a spliceosome (nuclear) RNA (Figure 2). Attempts to support our fractionation findings via complementary approaches, MS2-GFP binding and FISH (Stellaris), were unsuccessful as the first approach revealed a non-specific (i.e., similar to the reverse-complement transcript) enrichment in nuclear speckles and the second, no detectable signal. As an alternative, we took advantage of algorithms that have been engineered to predict lncRNA localization. One such tool, LncLocator is a recently available tool that utilizes a deep learning approach on a large set of experimentally validated lncRNAs to predict subcellular localization (Cao et al., 2018; Gudenas and Wang, 2018). Subjecting *CARMAL*’s sequence alongside various lncRNA of known distribution to the algorithm predicts a cytoplasmic localization, in line with our findings (Supplementary Table S1). Interestingly, the likelihood of nuclear localization remained substantial, on par with MALAT1, a lncRNA that was reported to shuttle between the nucleus and cytoplasm in a cell cycle dependent manner (Yang et al., 2013; Zhang et al., 2017).

**FIGURE 3:**
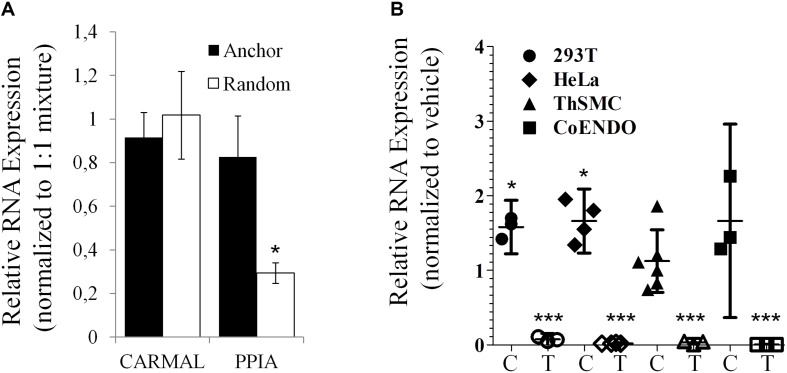
*CARMAL* is polyadenylated and stable. **(A)** *CARMAL* and *PPIA* levels were determined on cDNA prepared from HEK293T RNA in the presence of either random hexamer primer (poly(A) tail independent), oligodT anchor primer (poly(A) tail dependent) or a 1:1 mixture of both (standard condition), to which values are normalized. Results represent the mean of 3 biological replicates (±SD). **(B)** *CARMAL* (filled symbols; C) and *TRIB1* (an unstable mRNA control; matching empty symbols; T) expression levels measured in cells treated with either Actinomycin D or vehicle DMSO) only. Values represent the ratio of the relative RNA abundance (relative to *PPIA*) in Actinomycin D treated cells over the vehicle treated cells. Data from three to five biological replicates are shown (±95% CI). *p < 0.05. Changes for *TRIB1* were all highly significant (***p < 0.005).

Presence of a poly(A) tail was tested next as some lncRNA, particularly those arising from transcriptional “noise” or enhancer sequences have been reported to contain no poly(A) tail (Liu, 2017). While GTEx data were derived from RNA enriched by poly(A) bead isolation, the relatively low abundance reported by GTEx could reflect a substantial loss of a non-poly(A) population during isolation. To directly test for the presence of a poly(A) tail in *CARMAL*, cDNA synthesis reactions performed in the presence of either oligo(dT) anchors or random hexamers were performed; *CARMAL* does not contain internal poly(A) stretches that could promote internal primer binding and extension. Similar conversion to cDNA was obtained with oligodT anchor or random hexamers, pointing to the presence of a poly(A) tail in the bulk of the population (Figure 3). Addition of a poly(A) tail occurs co-transcriptionally with RNA polymerase II transcription, which is sensitive to Actinomycin D (ActD). Inclusion of Act D (for up to 5 h) did not reduce, indeed in some cells slightly increased, *CARMAL* levels, indicating that the RNA is basally stable and might be further increased via post-transcriptional processes upon ActD inhibition (e.g., by interfering with miRNA-mediated destabilization).

### Deletion of *CARMAL* Affects Flanking Gene Expression

**FIGURE 4:**
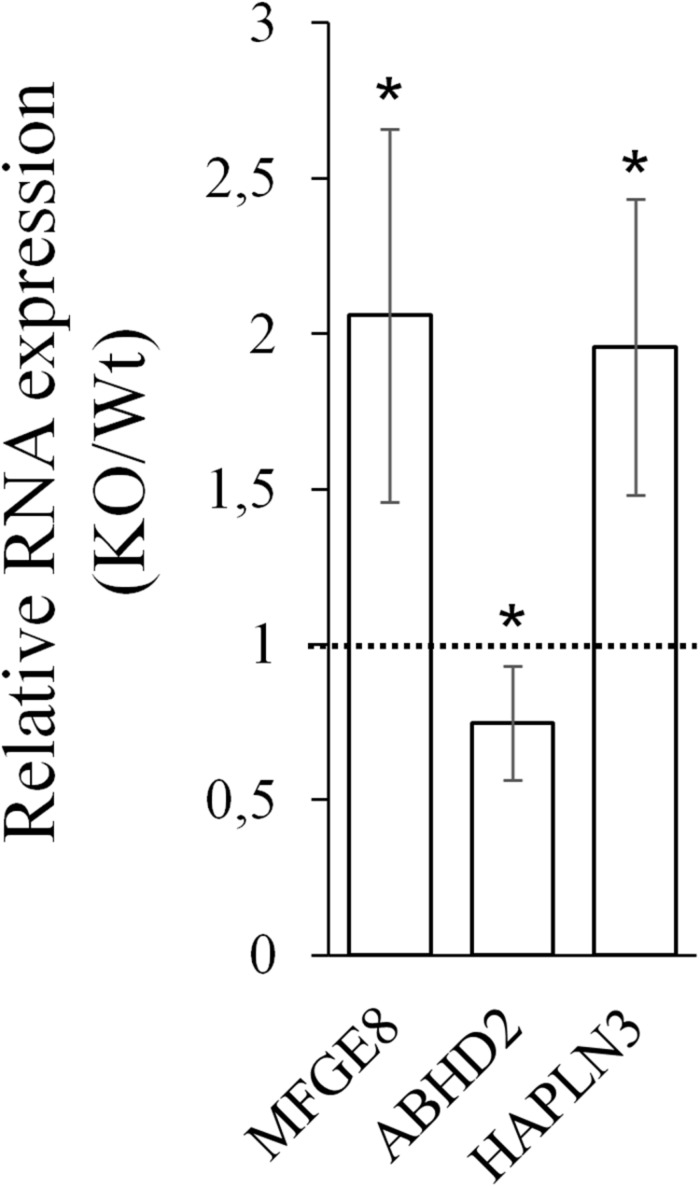
*CARMAL* deletion leads to increased *MFGE8* and *HAPLN3* but reduced *ABHD2*. RNA isolated from HEK293T, either Wt or harboring a CRISPR-Cas9-mediated *CARMAL* deletion (KO) were isolated and analyzed by qRT-PCR. Bars represent the average of eight (*MFGE8/ABHD2*) or four (*HAPLN3*) biological replicates (±95% CI). All changes between the KO and Wt were statistically significant (*p* < 0.05).

Next, *CARMAL* requirement was directly addressed by CRISPR mediated KO targeting of a ∼420 bp deletion spanning part of the promoter region as well as exon 1 of CARMAL in HEK293T cells. We previously demonstrated that the region proximal to the transcription initiation region acted as a promoter in a reporter assay (Soubeyrand et al., 2019a). Abrogation of *CARMAL* expression was first confirmed by qPCR using primers targeting distinct exon combinations (Supplementary Figure S4). The local impact of the disruption was examined first since we previously demonstrated effects of the abutting CARMA region on *HAPLN3* and *MFGE8* in HuH-7 (Soubeyrand et al., 2019a). Deletion of the *CARMAL* exon 1 region resulted in increased *MFGE8/HAPLN3* and reduced *ABHD2* consistent with a local regulatory function for *CARMAL* (Figure 4). Thus *CARMAL* may, together with the abutting CARMA genomic region, be involved in regulating *MFGE8* levels.

### Suppression of *CARMAL* Leads to a Modest Increase in *MFGE8*

**FIGURE 5:**
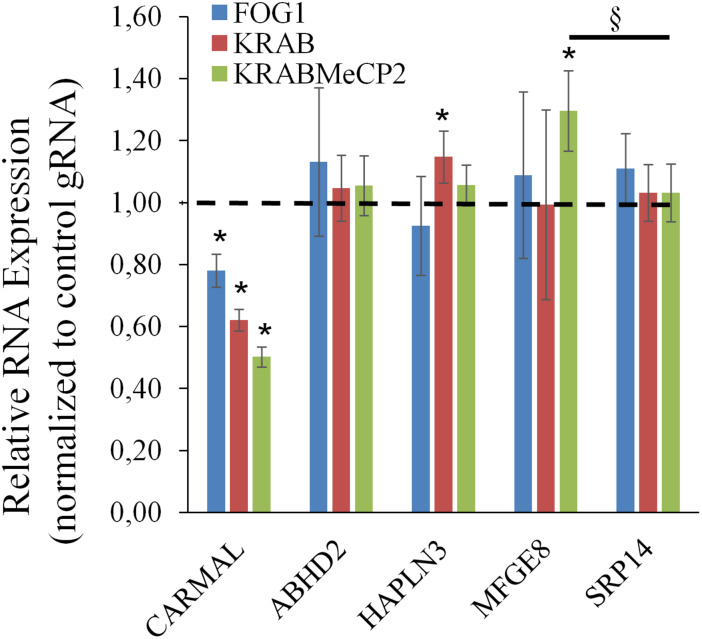
*CARMAL* suppression by KRABMeCP2-dCas9 is associated with increased *MFGE8* levels. HEK293T cells were transfected for 72 h with the indicated CRISPRi fusion constructs together with one of three distinct gRNA or a control gRNA (tracrRNA + linker only) expressing constructs. For each biological replicate, inhibition (vs trcrRNA control) values obtained using each gRNA were averaged. Values represent the average of three distinct biological replicates (±95% CI) *, <0.05 vs CRISPRi/trcrRNA only; ^§^*p* < 0.05 vs normalized *SRP14* values (one-way ANOVA).

The above findings pointed to a functional link between *CARMAL* and *MFGE8*, with *CARMAL* possibly contributing to the suppression of *MFGE8* under basal conditions. This could arise either as a result of loss of normal *CARMAL* expression or disruption of the gene locus *per se*. To test the contribution of the transcript to *MFGE8* expression, *CARMAL* was first targeted using antisense oligonucleotides. This analysis proved inconclusive as CARMAL was resistant to ASO treatment (Supplementary Figure S5). As an alternative approach, CRISPRi was used. We previously demonstrated that a KRAB derivatized inactive (dCRISPR) version had no measurable impact on local gene expression when targeted to the promoter region of *CARMAL*, despite achieving a ∼30% reduction in *CARMAL* expression (Soubeyrand et al., 2019a). Recently however, novel KRAB derivatives with improved inhibitory potential have been reported (Yeo et al., 2018). Using three distinct repressors (including KRAB for comparison) in combination with one of three distinct gRNA designed against intron 1 of CARMAL, we achieved 40–60% suppression of *CARMAL*. Interestingly, there was a statistically significant, albeit modest, increase in *MFGE8* when the most potent repressor (dCas9-KRAB-MeCP2) was used (Figure 5). Of note, with the exception of *CARMAL*, among the transcripts tested, only *MFGE8* was significantly affected relative to *SRP14*.

### Increased *CARMAL* Abundance and Expression Do Not Affect MFGE8

**FIGURE 6:**
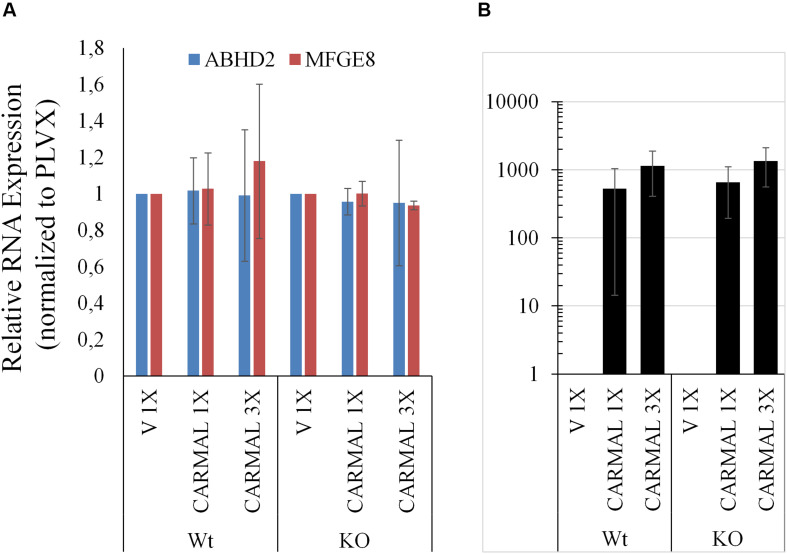
*CARMAL* overexpression affects neither *ABHD2* nor *MFGE8* in HEK293T. **(A)** Cells Wt or deleted for *CARMAL* (KO), were transduced with lentiviruses expressing *CARMAL* or control vector (V) for 48 h, were processed for qRT-PCR analysis. **(B)** *CARMAL* expression relative to vector infected (V = 1) controls. Results represent the means of three experiments (±SD).

Having examined the effects of *CARMAL* reduction, overexpression was investigated next. We previously demonstrated that a mild (∼4 X) upregulation of *CARMAL* via CRISPRa (VP160) had no effects on local gene expression, including *HAPLN3* and *MFGE8* (Soubeyrand et al., 2019a). These findings were confirmed in a new set of CRISPRa experiments using a more potent activator (SP-dCas9-VPR) and two distinct gRNAs (data is publicly available as GSE142097). In contrast to CRISPRa, transducing *CARMAL* led to a strong increase in *CARMAL* abundance (Figure 6B) which nonetheless failed to impact *MFGE8* or *ABHD2* (Figure 6A). Thus, these results agree with the CRISPRa data insofar that they demonstrate that higher *CARMAL* levels affect neither *MFGE8* nor *ABDH2* in Wt cells. In addition, the observation that the introduction of *CARMAL* increases neither transcript in *CARMAL* KO cells suggests that the *CARMAL* transcript is neither needed nor sufficient to maintain a steady-state level of *MFGE8/ABHD2*. Lastly, since the introduction of *CARMAL* does not reduce *MFGE8/ABHD2* levels in either cell type, the *MFGE8* upregulation seen in the KO and the CRISPRi experiments cannot be explained by the removal or reduction of a destabilizing influence of *CARMAL* on *MFGE8*.

### Transcriptome-Wide Impacts of *CARMAL* Deletion

Analyses thus far pointed to a likely role of the chromatin region in the regulation of local targets, but did not examine putative genome-wide roles of the *CARMAL* locus. Indeed, any impact *CARMAL* transcripts might have on local transcription is likely to be mediated indirectly via a feed-back loop involving the cytoplasm since our fractionation results are more in line with a cytosolic role. With the rational that this guilt-by-association approach might provide biological insight, transcription array results from *CARMAL-*deleted and control cells were contrasted, looking for emerging genome-wide patterns. *CARMAL* deletion resulted in changes in the abundance of 1.6% (Linear fold change >1.5, *p* < 0.05) of several transcripts, with a slight non-significant (Fisher’s *p* = 0.10) bias toward upregulation (433 vs 366). In line with the earlier qPCR results, *CARMAL* deletion led to an increase in *MFGE8* (1.34-fold, *p* = 0.02) and a reduction in *ABHD2* (−1.67-fold, *p* = 0.003). While *HAPLN3* levels increased on average, in agreement with the qPCR results, changes did not reach statistical significance (1.12-fold, *p* = 0.29).

### Over-Representation Analysis of Transcripts Impacted by *CARMAL* Deletion

**FIGURE 7:**
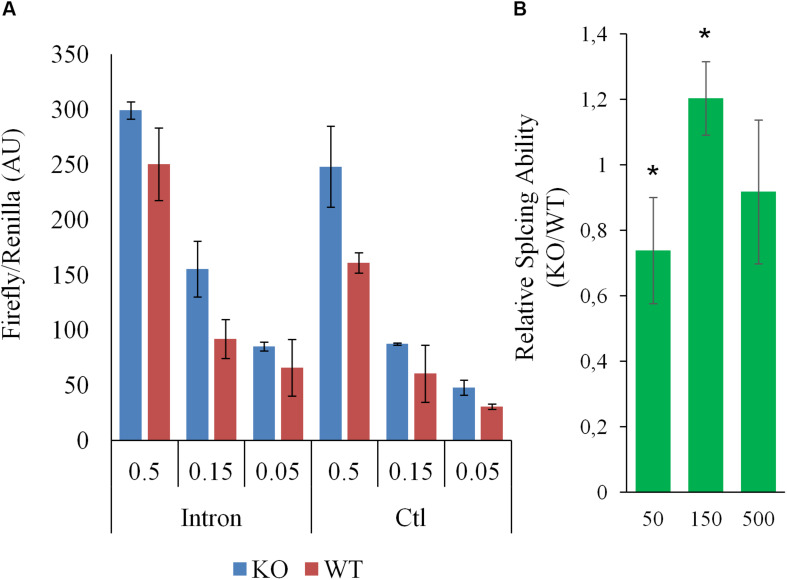
Splicing in WT and *CARMAL* deleted HEK293T differ. Wild-type (WT) or *CARMAL* deleted cells in 24 well plates were transfected for 24 h with variable amounts (μg) of either an intron-containing luciferase construct or an intronless control [CMV-LUC2CP/intron/ARE and CMV2-LU2CP/ARE (Addgene)], together with 1% of Renilla internal control. Four independent experiments were performed over a 2 week period. **(A)** Representative experiment is shown (average of two distinct DNA preparations ± SD). **(B)** Average ability to process intron (intron/intronless values) of the CARMAL deleted cells relative to the Wt controls, over four experiments (±SD). Values per dose (in ng per well) of transfected DNA are shown.

To help pinpoint a possible transcriptome-wide trend, over-representation analyses, which hinge on the enrichment of a subset of genes (significantly impacted array hits) within a larger list (entire array list) were first performed. The analysis, focusing on nominally significant hits (*p* < 0.05, >1.5-liner fold change) and the Reactome and KEGG pathway databases [via WebGestalt (Subramanian et al., 2005; Liao et al., 2019)], failed to detect FDR significant enrichment. The analysis was repeated with a more stringent hit list (FDR significant hits only; 64 unique Entrez Gene IDs) with similar results. This analysis relied on pathway information as defined by Reactome and KEGG and did not examine other informative gene properties (biochemical features, protein modification etc…). To obtain a more comprehensive view of the genes impacted by the deletion, nominally significant hits were subjected to DAVID (Database for Annotation, Visualization and Integrated Discovery), specifically looking for additional properties. Several (9) categories were significantly (FDR <0.05) enriched within this list, including several UniProt tags (UP_KEYWORDS) (Table 1). Interestingly, alternative splicing was the most statistically significant hit by far, indicating that *CARMAL* deletion disproportionally affects alternatively spliced transcripts although the overall enrichment was low (1.11-fold). In addition, similar numbers of upregulated and downregulated genes were observed in the enriched population (Fisher’s = 0.52). A putative role in splicing was interrogated via a reporter assay consisting of luciferase constructs harboring an internal intron and its matching control (Younis et al., 2010). The assay indeed revealed a difference between Wt and *CARMAL* KO cell (Figure 7).

**TABLE 1:** Overrepresentation analysis of transcripts responding to CARMAL deletion.

**Category**	**Term**	**Count**	**List total**	**Pop hits**	**Pop total**	**Fold enrichment**	**PValue**	**BH FDR**
UP_KEYWORDS	Alternative splicing	1424	2492	10587	20581	1.110850234	7.29E-10	4.04E-07
UP_KEYWORDS	Phosphoprotein	1111	2492	8246	20581	1.112728376	6.48E-07	0.00018
UP_KEYWORDS	Cytoplasm	678	2492	4816	20581	1.162683877	1.56E-06	0.000288
UP_KEYWORDS	Nucleus	727	2492	5244	20581	1.144959599	5.59E-06	0.000774
UP_KEYWORDS	Golgi apparatus	137	2492	812	20581	1.393422993	4.57E-05	0.005048
UP_KEYWORDS	Acetylation	480	2492	3424	20581	1.157779661	0.000146	0.013411
GOTERM_CC_DIRECT	GO:0005813~centrosome	82	2253	426	18224	1.556993388	4.47E-05	0.037525
GOTERM_CC_DIRECT	GO:0005802~trans-Golgi net.	34	2253	136	18224	2.022192632	9.27E-05	0.03892

*Nominally affected transcript were mapped by DAVID to 3275 Entrez IDs and analyzed for overrepresentation analysis (ORA). Category: Category output. Term: Term within the category identified by DAVID. Count: number of IDs matching the Term with the list of interest. List Total: total number of mappable IDs within list of interest. Pop Hits: number of IDs matching the Term in the background list; Pop Total: total number of mappable IDs in the background list. Enrichment: fold enrichment of the term in the list vs background. PValue: nominal *p* value. BH FDR: Benjami-Hochberg corrected False Discovery Rate.*

### Gene Set Enrichment, PADOG and Ingenuity Pathway Analysis

One limitation of over-representation analysis is that by focusing on statistically changed genes, it ignores the underlying magnitude and direction of the changes, which carry important biological information. To capture the dynamics of the affected pathways, two ranking analyses were used. First, Gene Set Enrichment Analysis (GSEA) of the entire ranked (fold-change) dataset was performed, again using Reactome mapped pathways via the WebGestalt interface (Table 2 and Supplementary Figure S6). This inquiry revealed that deletion of *CARMAL* was associated with a possible enrichment in chromatin remodeling in deleted cells, with an FDR significant signal for histone acetylation. To further mine the dataset, PADOG (Pathway Analysis with Down-weighting of Overlapping Genes) was used (Tarca et al., 2012). A unique feature of PADOG is that it weighs genes according to their presence across multiple pathways, favoring less “frequent” (i.e., more pivotal) genes. The analysis resulted in the identification of 28 nominally (*P* < 0.05) and 11 FDR (*q* < 0.05) significant KEGG pathways, albeit with no salient uniting theme (Supplementary Table S2).

**TABLE 2:** Gene set enrichment analysis of *CARMAL* KO cells implicates chromatin remodeling.

**Gene set**	**Description**	**Size**	**Leading edge number**	**ES**	**NES**	**FDR**
R-HSA-3214847	HATs acetylate histones	128	36	0.21176	2.4582	0.046996
R-HSA-2559582	Senescence-Associated Secretory Phenotype	96	46	0.23162	2.3856	0.054247
R-HSA-3214858	RMTs methylate histone arginines	68	31	0.27329	2.4868	0.063334
R-HSA-201722	Formation of the beta-catenin:TCF complex	79	29	0.23953	2.2332	0.076631
R-HSA-3214815	HDACs deacetylate histones	81	33	0.23395	2.285	0.080407
R-HSA-3214842	HDMs demethylate histones	42	29	0.30264	2.2356	0.087934
R-HSA-8878171	Transcriptional regulation by RUNX1	220	60	0.14351	2.0917	0.088852
R-HSA-73728	RNA Polymerase I Promoter Opening	51	24	0.28042	2.169	0.09081
R-HSA-3247509	Chromatin modifying enzymes	254	85	0.13944	2.1375	0.091122
R-HSA-4839726	Chromatin organization	254	85	0.13944	2.1375	0.091122

*GSEA was used to interrogate a list of genes ranked according to the change in expression magnitude observed between the *CARMAL* KO and controls. The submitted list contained 29590 IDs of which 25339 IDs mapped to Entrez Gene IDs. Among these, 9818 IDs mapped to functional Reactome categories. Results shown include all hits with FDR <0.1. Gene Set: Reactome identifier; Description: Reactome synonym. Size, number of molecules in the identifier; Leading Edge number: number of genes populating the gene set that drive the enrichment signal; ES: enrichment score; NES: normalized enrichment score; FDR: false discovery rate to correct for multiple hypothesis testing. Analysis was performed via WebGestalt.*

To help pin down affected pathways, a topology-based analysis was used. Ingenuity (IPA) uses positional information within curated pathways to garner further information, including possible regulators. Ingenuity also uses its own curated “canonical” pathways that were interrogated first. The analysis identified multiple pathways that were nominally affected although none reached FDR (B-H corrected) significance (Supplementary Table S3). Interestingly the top identified pathway (Leucine Degradation I) overlapped with a PADOG FDR significant hit (valine, leucine and isoleucine degradation). The negative z-score suggests a reduced ability to degrade leucine in *CARMAL* deleted cells. Upstream Regulator Analysis (URA) was then performed to predict candidate regulators compatible with the (nominally) differentially expressed genes. This analysis identified patterns consistent with the implication of several regulators or conditions (Table 3 and Supplementary Table S4). Of particular interest, changes resembled those ensuing from the treatment with three inhibitors (tazemetostat, decitabine, SP2509) targeting (de)methylation processes, indicative of possible repercussions of *CARMAL* deletion on genome-wide methylation. The most statistically significant regulator was SBDS (Shwachman-Bodian-Diamond Syndrome), a regulator of ribosomal maturation (Weis et al., 2015). Transcription array data however, indicated that *SDBS* was not significantly affected at the transcript level. Possible post-transcriptional regulation was assessed by Western blotting, that revealed no significant difference in SBDS level across both cell populations (Supplementary Figure S7).

**TABLE 3:** Upstream regulator analysis by ingenuity.

**Upstream regulator**	**Expr fold change**	**Molecule type**	**Predicted activation state**	**Activation *z*-score**	***p*-value of overlap**	**B-H corrected *p*-value**
SBDS	–	Other	–	−	4.57E-07	0.00291
NUPR1	−1.34	Transcription regulator	–	1.949	7.69E-06	0.0245
ERBB2	–	Kinase	–	–0.761	1.78E-05	0.0245
TCF4	–	Transcription regulator	Inhibited	–2.075	1.91E-05	0.0245
tazemetostat	–	Chemical drug	–	1.432	1.93E-05	0.0245
SP2509	–	Chemical reagent	–	1.542	3.26E-05	0.0308
decitabine	–	Chemical drug	–	1.048	3.39E-05	0.0308

*Transcripts whose expression was significantly (*p* < 0.05) changed between KO and WT cells were subjected to IPA to identify possible impacted regulators. Only FDR significant hits are shown. A complete list of Regulators (including molecules in datasets) is included as Supplementary Table S3.*

## Discussion

This work examines a reported but largely uncharacterized lncRNA locus, *CARMAL/RP11-326A19.4.* In a previous study, we showed the corresponding transcript to be somewhat conserved but only superficially explored its function. The current study explores the functional aspects of *CARMAL*, both locally and genome-wide. At the local level, we show that transcript levels of gene neighbors are affected by targeting the *CARMAL* locus, although the mechanisms require further clarification. We demonstrate that *CARMAL* is enriched in the cytosol; while attempts to support our *CARMAL* fractionation data via microscopic approaches were unsuccessful, cytosolic localization was supported by bioinformatic evidence. Of note, a recent large scale bioinformatic analysis indicates that lncRNA subcellular localization is largely conserved across cell types (Gudenas and Wang, 2018). Notwithstanding its classification as a lncRNA, the possibility remains that the *CARMAL* transcript encodes bioactive short peptides, which in turn may regulate nuclear function. Indeed, examination of ribosome profiling data from GWIPS-viz (Michel et al., 2014) indicated a weak ribosome association to the identified exons in HEK293T cells, particularly around exon 1 (Supplementary Figure S8). However, in accordance with its classification as a lncRNA, Protein BLAST or the Web CD-Search tool^1^ failed to identify any sequence or domain pattern in the predicted amino acid sequences, casting doubt on the biological relevance of any putative peptide output (Supplementary Figure S8).

Primary differentiated cell models are inadequate as experimental models for CRISPR-related experiments due to their poor transfectability and finite proliferative capacity. Our choice of HEK293T to explore *CARMAL* function was based on several criteria: a human origin, significant expression of *CARMAL*, euploidy over the entire CARMA region (as per ENCODE/HAIB data), high transfection efficiency compatible with large CRISPR plasmids, a successful history of CRISPR editing, as well as robust growth. Use of HEK293T comes at the expense of a reduced *a priori* translatability to normal tissues and some findings will have to be validated in cells more pertinent to CAD. No cellular model is perfect however. For one, the normal arterial vasculature consists of several cell types and while smooth muscle cells constitute the bulk of the cell mass, other cells populations may contribute to *CARMAL* expression than their relative abundance (Roostalu and Wong, 2018). In addition, smooth muscle cells are phenotypically plastic and adapt to their environment by adjusting their transcriptional profile to *ex vivo* conditions (Alexander and Owens, 2012).

*CARMAL* was expressed at relatively low levels in HEK293T and in two vascular cell models. These results are in line with GTEx quantification in arteries (in the low transcripts per million range), indicating that low expression is a feature of its biological role. Low expression contrasts with the observation that the *CARMAL* promoter region is active in promoter trapping assays (Soubeyrand et al., 2019a) suggesting that the promoter region is under tight repressive control in its native environment. Interestingly common genetic variants (e.g., rs2083460) that are expression quantitative trait loci (eQTL) for *CARMAL* show eQTL effects for *MFGE8* expression in coronary vessels (GTEx), indicating that the region is under concerted regulation in vascular tissues. Thus polymorphisms that associate with *MFGE8* expression may exert this effect, in part, by altering *CARMAL* expression. Since reducing *CARMAL* expression increases *MFGE8*, a normal function of *CARMAL* might be to moderate *MFGE8* expression, which we previously demonstrated correlated with CAD (Soubeyrand et al., 2019b). One related question of interest is the impact of CAD and its progression on vascular expression of *CARMAL* and *MFGE8*. As *CARMAL* is not conserved in rodent models, answering that question will require interrogating diseased and control coronary tissue from human cohorts.

Upregulation of *MFGE8* required substantial suppression of the *CARMAL* locus, achieved by one of the 3 types of KRAB derivatives tested. As all three CRISPRi effectors are targeted to the same (intronic) regions of *CARMAL*, and thus are expected to similarly perturb local chromatin, additional factors are likely to be at work. One simple interpretation is that *CARMAL* must be reduced by >∼60% in order to affect *MFGE8*, and thus that a minimal level of *CARMAL* >∼40% is needed for its expression. This latter explanation however, fails to account for the inability of transduced *CARMAL* to increase (i.e., restore) *MFGE8* in KO cells. Alternatively, the type of KRAB derivative may impart changes to the region more conducive to *MFGE8* transcription, independent of its impact on *CARMAL* expression although it is unclear how a repressor such as KRAB-MeCP2 could activate local transcription directly. In summary, these findings indicate that *CARMAL* transcription represses *MFGE8* under basal conditions and point to the importance of the *CARMAL* locus proper, rather than its transcript, in this process.

Ontology analysis uncovers potential differences in RNA splicing following *CARMAL* deletion. This finding is further supported by direct evidence from a reporter assay demonstrating a non-linear difference in the ability of these cells to effect splicing. Unfortunately the transcript arrays used to interrogate our differential expression samples do not carry splicing variants information. Beyond supporting a possible role in splicing, how this dose-dependence in splicing ability translates to the natural splicing environment is unclear. Additional experiments will be needed to first ascertain whether *CARMAL* is directly involved or this represents a cell adaptation to the genetic editing or a stochastic genetic drift.

Impact analysis (URA) reveals expression changes consistent with a contribution of *SBDS* as a result of *CARMAL* deletion. Through URA we previously identified *HNF4A* as downstream effector of *TRIB1* (Soubeyrand et al., 2017). The evidence for *SBDS* involvement is derived from RNA expression data derived from *SDBS* siRNA experiments performed in HEK293T, the main cell type used in this study. Given that our expression array data indicate that *SBDS* levels are unaffected by *CARMAL* deletion, *CARMAL* may regulate SBDS post-transcriptionally. Although we did not find a significant impact on SBDS protein level, the absence of *CARMAL* may affect its function via changes in post-translational modifications (PTM). Multiple PTM within SBDS have been identified via proteome-wide analyses, but further validation and functional studies are required^2^. Dysfunctional SBDS can lead to the Shwachman-Bodian-Diamond syndrome, a recessive disease defined by a complex suite of clinical features including pancreatic dysfunction, short stature and leukemia (Boocock et al., 2003; Warren, 2018). In human cohorts, an intronic variant in *SBDS* (rs12667745) is associated with peripheral vascular disease and respiratory disorders (UKBB data accessed through phenoscanner^3^). At the cellular level, SBDS has been identified as a key regulator of late (cytoplasmic) ribosomal assembly and has been reported to shuttle across the nuclear membrane (Finch et al., 2011; Orelio et al., 2011; Weis et al., 2015). Moreover *SBDS* may play multiple roles. For instance, nuclear forms of SBDS appear to contribute to the maintenance of telomere integrity (Liu et al., 2018). Given its low expression, *CARMAL* is not expected to play a major role in ribosomal maturation. A putative functional interaction between *CARMAL* and SBDS is likely restricted to other processes but remains an intriguing possibility that we are currently exploring.

## Methods

### Cell Culture, Transfection and Transductions

Lentiviral particles were generated using HEK293FT using pLVX, pSPAX2 and pMD2. G (from Addgene). Supernatants were filtered through 0.4 μM filters, titered using qPCR assays of viral cDNAs and stored at −80°C. HEK239T cells were transfected with expression plasmids using Lipofectamine 3000 [1:2:3 ratio of DNA (μg): P3000 (μl):lipofectamine 3000 (μl) reagent] or RNAiMax. Unless specified, 0.5 μg DNA was used per 200 mm^2^ and 70% confluent cells. Oligonucleotide transfection was performed using lipofectamine RNAiMax (ThermoFisher) and 10 nM final concentration.

### CRISPR-Mediated KO of CARMAL

Deletion was performed on HEK293T cells using gRNA flanking the promoter and exon 1 of CARMAL. The gRNA vector was derived from pCR2.0 by insertion of a U6 promoter-tracrRNA cassette from pX330-U6-Chimeric_BB-CBh-hSpCas9 (Cong et al., 2013). Single clones were obtained by fluorescent assisted cell sorting, were expanded and screened using a step-wise approach. Deletions were first screened by real time PCR looking for loss of signal over the deleted region, followed by a positive selection using primers spanning the deletion. Out of 100 screened clonal populations, bi-allelic deletion was observed in a single clone. Sanger sequencing of the clone revealed that one allele was deleted as intended while the other retained a short (∼80 bp) promoter fragment that was reintroduced in the reverse orientation. See Supplementary Data Sheet 3 for sgRNA sequence information.

### CRISPRi and CRISPRa

Plasmids used for CRISPRa and CRISPRi were obtained from Addgene. Specifically dCas9-KRAB-MeCP2 (#110821), dCas9-FOG1[N + C] (#100085), dCas9-KRAB (#110820); CRISPRa was a VPR derivative (SP-dCas9-VPR; #63798) (Chavez et al., 2015; O’Geen et al., 2015; Yeo et al., 2018). For a typical experiment, the dCAS9 plasmid was co-transfected at a mass ratio of 7:3 together with a sgRNA plasmid (pCRU6) expressing the single-guide RNA driven by a U6 promoter; for a 12-well plate format, wells were transfected with either 0.5 μg (CRISPRa) or 1 μg (CRISPRi). Transfections were performed for 48–72 h as indicated. Guide sequence information are detailed in Supplementary Data Sheet 3.

### PCR and qRT-PCR

For standard PCR analysis, DNA samples were analyzed using Terra PCR direct for 35–40 cycles as suggested by the supplier (Takara Bio). Quantitative (RT-) PCR analyses were used using the SYBR Green I Master Mix on a LightCycler 480 (Roche) and a 65–55°C touchdown over 45 cycles (30 s extension). For cDNA synthesis, mRNA was first isolated using the High Pure RNA Isolation Kit (Roche), followed by cDNA was synthesis using the Transcriptor First Strand cDNA Synthesis Kit (Roche) according to the supplier’s protocol, including a 5 min 65°C denaturation/annealing step and a 50°C (1 h) reverse transcription step. Unless mentioned otherwise, a typical reaction included 0.1–0.5 μg of RNA and a 1:1 mixture of an anchored oligo(dT)18 oligomer and random hexamers. For Droplet Digital RT-PCR, cDNA was first generated according to the standard RT protocol and mixed with QX200 ddPCR EvaGreen Supermix (Biorad) following the manufacturer’s protocol. Droplets were prepared using QX200 droplet generation oil on a QX200 Droplet Generator (Biorad) and subjected to PCR using a C1000 thermocycler (BioRad) as follows: 95°C for 5 min, followed by 45 cycles of 95°C for 30 s at a ramp rate of 2°C/s and 60°C 1 min extensions at a ramp rate of 2°C/s. Positive/negative droplets were counted by a QX200 Droplet Reader (BioRad). All primers are described in Supplementary Data Sheet 3.

### Transcriptomics

Transcriptome analyses were performed at the TCAG (Toronto) using Human ST 2.0 arrays. All mRNAs were purified via High Pure RNA isolation spin columns (Roche). For Knock-out analysis, three distinct wild type populations were mixed and compared with the *CARMAL* deleted clone. RNAs harvested over four passages were processed individually for array analysis, for a total of eight analyzed samples. For CRISPRa, three independent transfections were analyzed. DataSets and experimental details can be found at the Gene Expression Omnibus: GSE142097 and GSE142098 for the activation and deletion datasets, respectively.

### RNA Fractionation Analysis

Samples were fractionated essentially as described previously (Rinn et al., 2007) and RNA therein isolated (High Pure RNA isolation kit, Roche). RNA (0.2–0.5 μg) was then converted to cDNA (First Strand RNA Transcriptor, Roche) and quantified by qRT-PCR. Nuclear enrichment was then quantified using the deltaCt method, subtracting the cytosolic from the nuclear signal Ct values (deltaCt). Nuclear enrichment was then 2ExpdeltaCt, with values over 1 reflecting respectively nuclear enrichment and below 1, cytosolic enrichment.

### Bioinformatic Analysis

Multiple approaches were used with the rational that coherent results emerging from complementary approaches should be more robust findings. DAVID (Huang et al., 2009) was used using default settings with the inclusion of Reactome and UP_Tissue datasets. The whole array list (Human ST 2.0) was used as background. WebGestalt GSEA analyses were performed using the default settings. GSEA is a rank based method that utilizes *a priori* defined gene set information (pathways) to identify the most impacted biological processes (Subramanian et al., 2005). Although related to GSEA (ranked and enrichment based), PADOG uses a weighting function that favors genes that tend to be enriched in particular gene sets and downweights genes that are shared across gene sets, with the rational that these genes play more central roles and are therefore more likely to impact any given pathway when perturbed. Moreover, PADOG uses a different gene-scoring method. The approach is more sensitive, possibly at the expense of reduced specificity (Nguyen et al., 2019). PADOG (3.1) was run in R (3.6) using default settings. For IPA (Ingenuity), a list of 48227 Affymetrix IDs were submitted, which were mapped to 30067 IPA addresses. Using a nominal (0.05 cutoff) filter resulted in 3035 molecules that were analyzed by core analysis using the default settings.

## Data Availability Statement

The datasets generated for this study can be found in the Gene Expression Omnibus (GEO): GSE142097 and GSE142098.

## Author Contributions

SS and RM planned and designed the research. SS, PL, H-DH, and AT conducted the experiments. TA provided logistical assistance. MN conducted the PADOG analysis. SS and RM wrote the manuscript. All authors contributed to the article and approved the submitted version.

## Conflict of Interest

The authors declare that the research was conducted in the absence of any commercial or financial relationships that could be construed as a potential conflict of interest.

## Footnotes

1 https://www.ncbi.nlm.nih.gov/Structure/bwrpsb/bwrpsb.gi

2 https://www.phosphosite.org/proteinAction?id=17958&showAllSites=true

3 http://www.phenoscanner.medschl.cam.ac.uk/
