# Supplementary material for: CARMAL Is a Long Non-coding RNA Locus That Regulates MFGE8 Expression
Source: Front Genet. 2020 Jun 17;11:631. doi: 10.3389/fgene.2020.00631 (PMC7311772; doi:10.3389/fgene.2020.00631)
Supplement: Supplementary file 1 [file Data_Sheet_1.PDF]

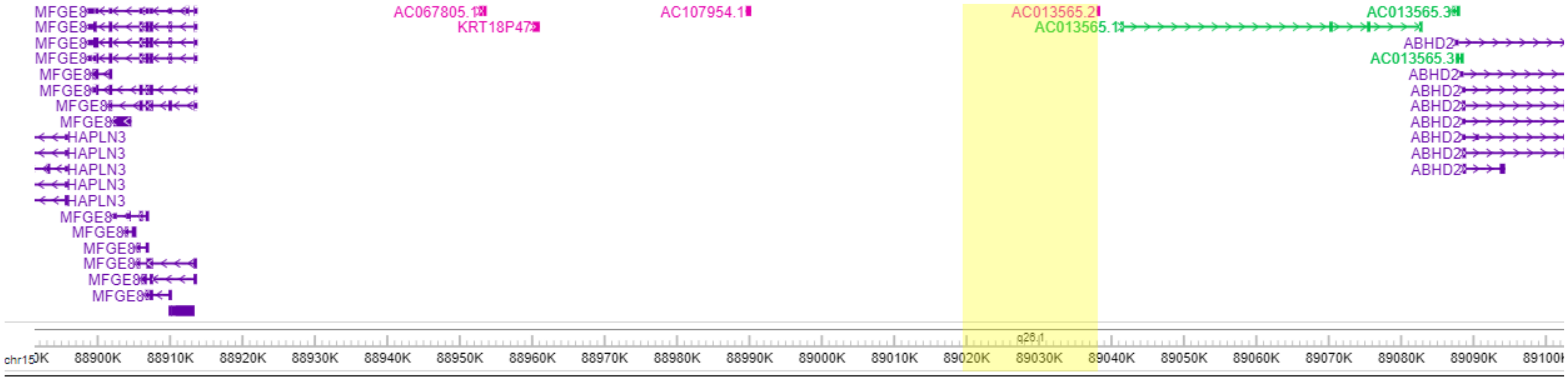

Figure S1. Location of *RP11-326A19.4/AC013565*. A, WashU epigenome browser snapshot highlighting the genes (genecodeV29, comprehensive dataset) surrounding the CAD associated region (yellow, corresponding to chr15:89,019,583-89,038,157) and its proximity to *CARMAL (*AC013565*)*. Genes colored in green represent lncRNA while genes in purple represent protein coding genes. Genes in pink represent pseudo genes.

Figure S2. CAD associated SNP rs2083460 is associated with changes in RP11-326A19.4 expression in the arteries. Data from GTEx (V7). Normalized effect size on the left and individual violin plots below.

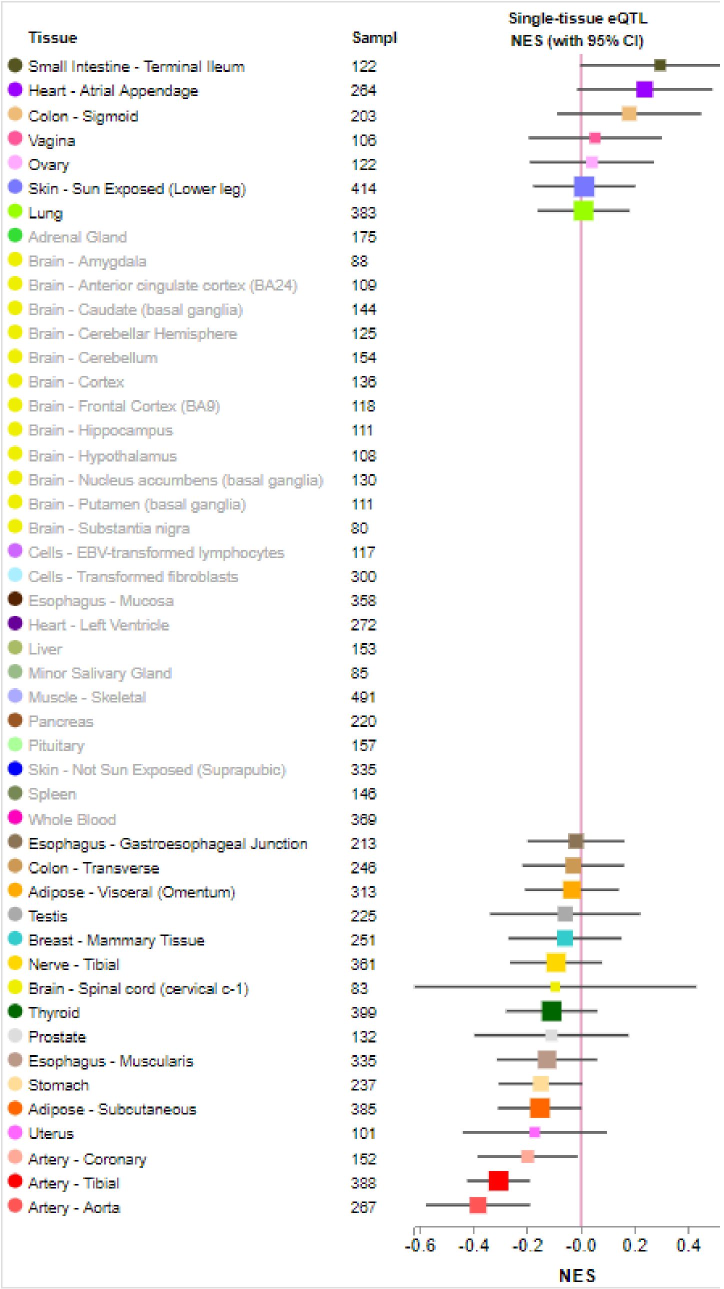

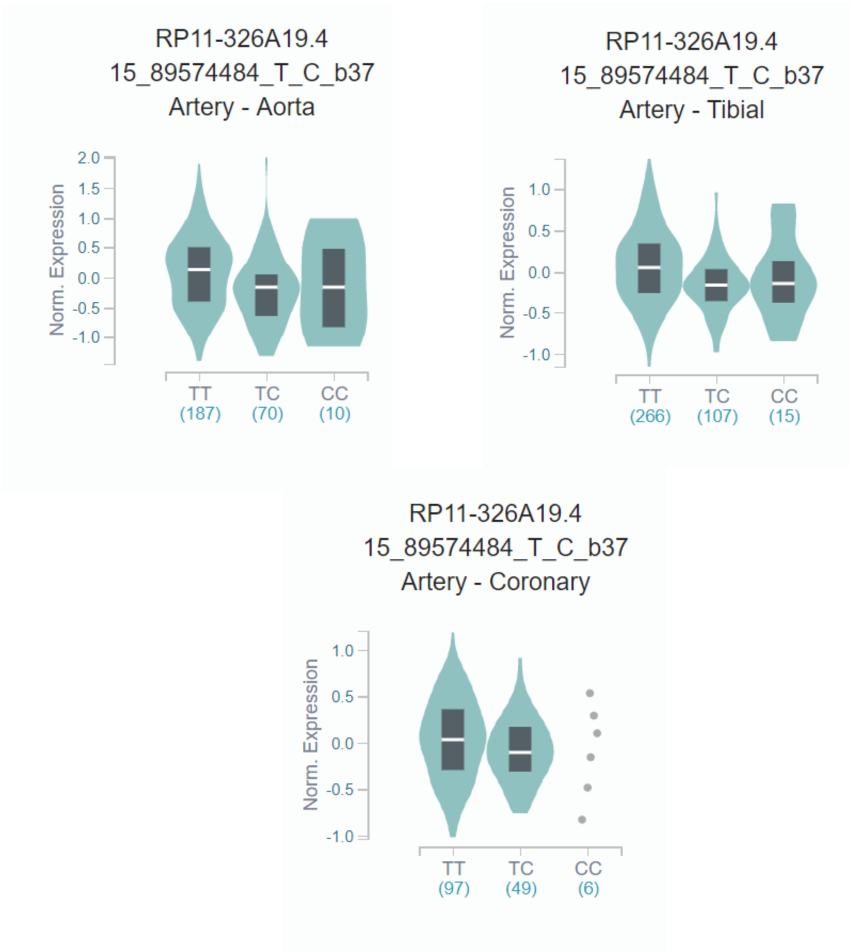

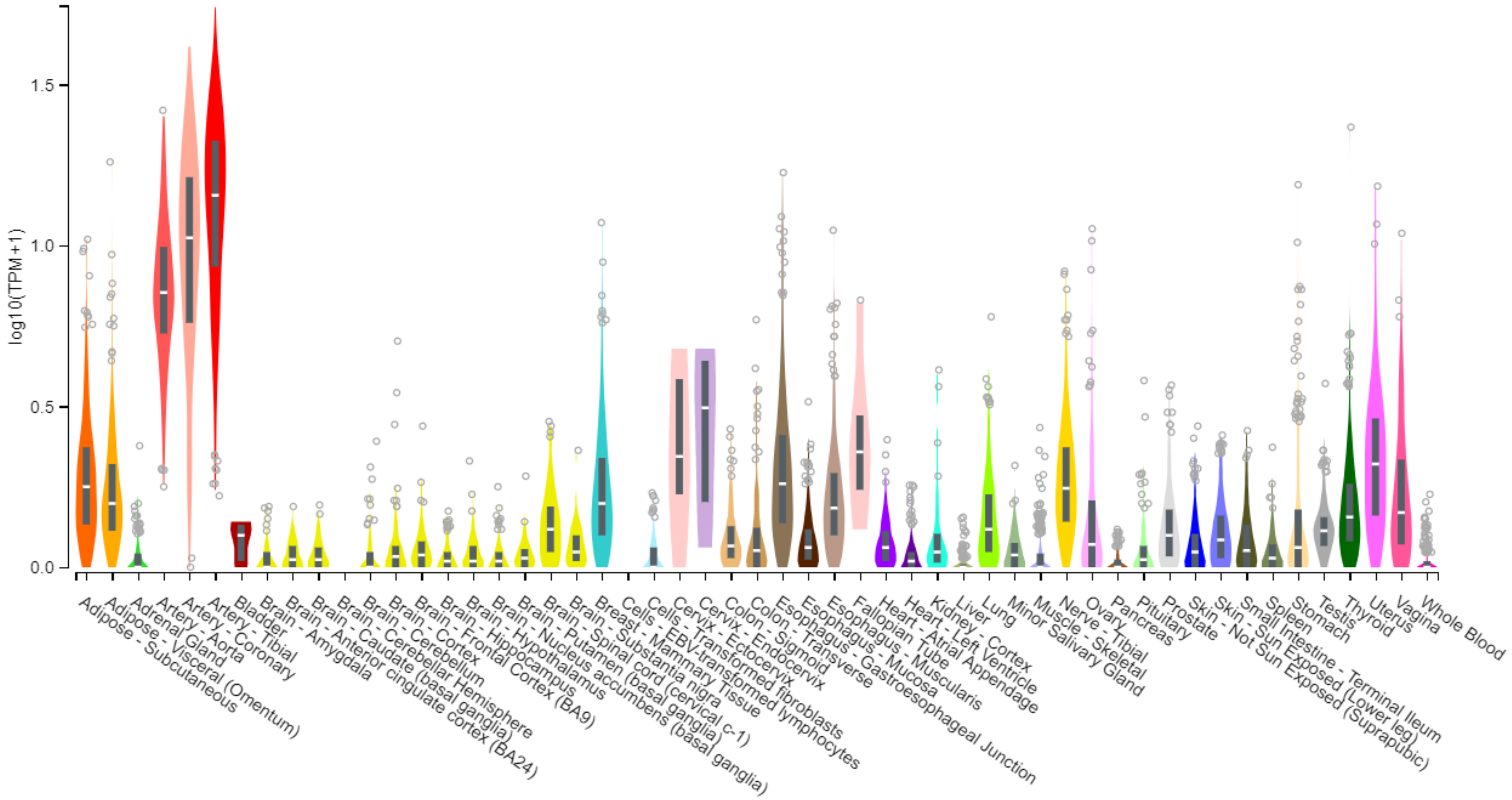

Figure S3. Expression profile of *RP11-326A19.4/AC013565*. Expression in various tissues and biological compartments as determined by the GTEx consortium.

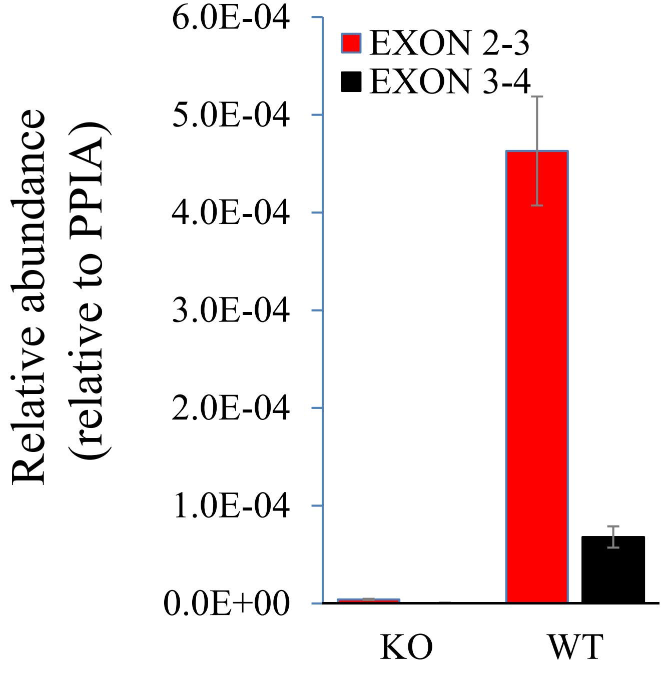

Figure S4. Validation of array results by qRT-PCR**.** RNA samples analyzed by expression array were in turn interrogated by qRT-PCR**.** Deletion of exon 1 of CARMAL abrogates its expression. Deletion of the promoter and exon 1 of *CARMAL* resulted in loss of expression of downstream exons of *CARMAL*. Pairs of primers spanning introns and recognizing either exons 2 and 3 or exons 3 and 4 were used as indicated. Bars represent the average of 3 biological replicates (±S.D.).

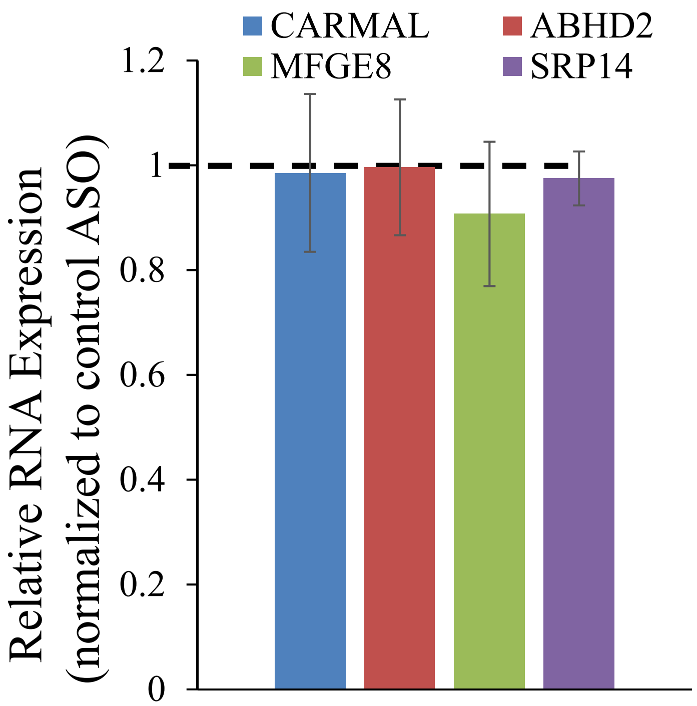

Figure S5. *CARMAL* is resistant to antisense oligonucleotides. Antisense oligonucleotide treatment. HEK293T cells were treated for 48 h with one of 3 ASO (one ASO per treatment) or a control ASO. For each experiment, results from the 3 CARMAL ASO were normalized to the control ASO and averaged . Data represent the average of 3 distinct biological replicates (± 95% C.I.)

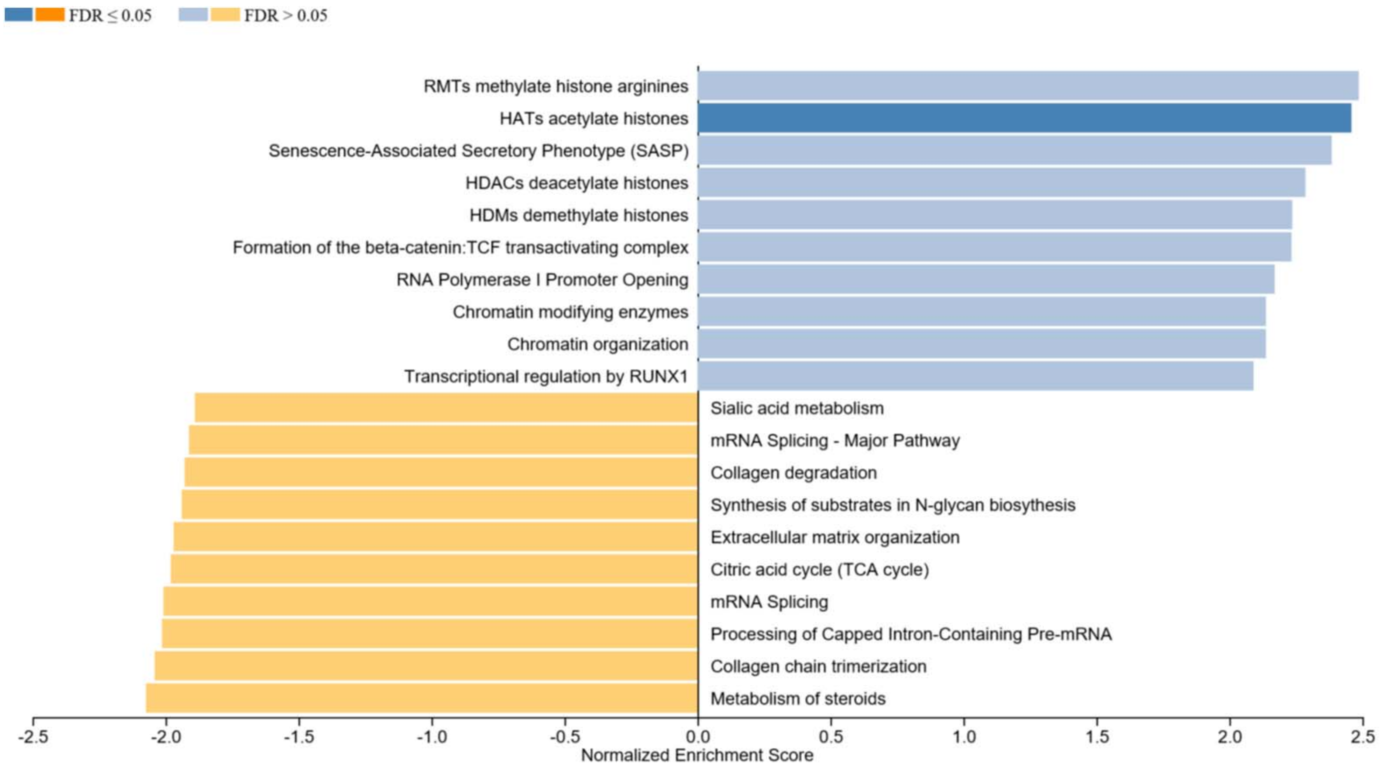

Gene Symbol	Fold-change (KO/Wt)	Nominal p value
HIST1H3B	1.81	0.03
HIST1H4H	1.54	0.33
HIST1H4B	1.45	0.09
HIST1H2AE	1.44	0.02
KAT2B	1.44	0.001
HIST1H3A	1.43	0.02
KAT6A	1.43	0.002
HIST1H2BN	1.41	0.02
EP400	1.39	0.05
HIST1H2BB	1.36	0.28
HIST1H3E	1.36	0.02
TRRAP	1.36	0.02
HIST1H3G	1.31	0.29
HIST1H2BG	1.29	0.36
ATXN7	1.28	0.02
EPC1	1.27	0.01
HIST1H2BF	1.27	0.13
NCOA2	1.27	0.04
KAT5	1.26	0.2
HIST1H2AH	1.25	0.66
HIST1H2BD	1.25	0.09
BRPF1	1.24	0.12
BRPF3	1.24	0.25
HIST1H3I	1.22	0.01
ELP3	1.21	0.09
KANSL1	1.21	0.17
RUVBL1	1.21	0.36
HIST1H2AL	1.2	0.02
HIST1H3F	1.2	0.32
ATXN7L3	1.19	0.16
HIST1H2BK	1.19	0.46
HIST1H3J	1.19	0.41
KANSL2	1.19	0.06
JADE2	1.18	0.64
MCRS1	1.18	0.3
MSL2	1.18	0.02

Figure S6. Processes most impacted by CARMAL deletions identified by Gene Set Enrichment Analysis. Only "HATs acetylate histones" was FDR significant. Right, leading edge genes i.e. genes whose expression differs between CARMAL deleted and control controls and populate the gene set "HATs acetylate histones". Fold change and nominal p values are also included; data bars (p values) are overlaid to facilitate visualization of statistically significant changes.

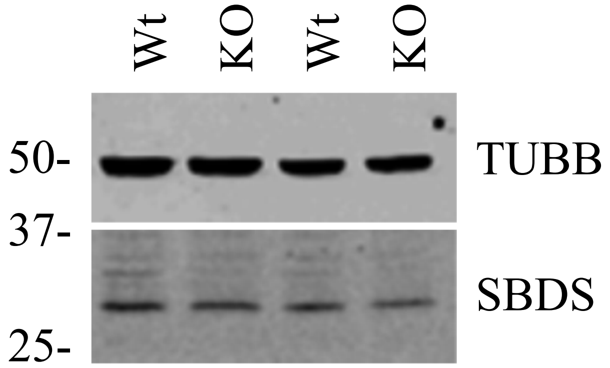

Figure S7. Representative SBDS Western blot showing no significant difference between Wt and *CARMAL* KO cells. Three distinct dilutions (15µg and 5 µg) lysate were analyzed by WB (12% SDS-PAGE gel). Relative intensities of the TUBB and SBDS signals were calculated at each dose, expressed relative to TUBB (SBDS/TUBB) and the corresponding values averaged to yield an average value per biologic. The analysis was performed for 3 distinct biological repeats, over 3 distinct doses, and revealed non significant differences between the 2 populations, after correction for TUBB (KO/Wt: 1.0 ± 0.18, 95% C.I.).

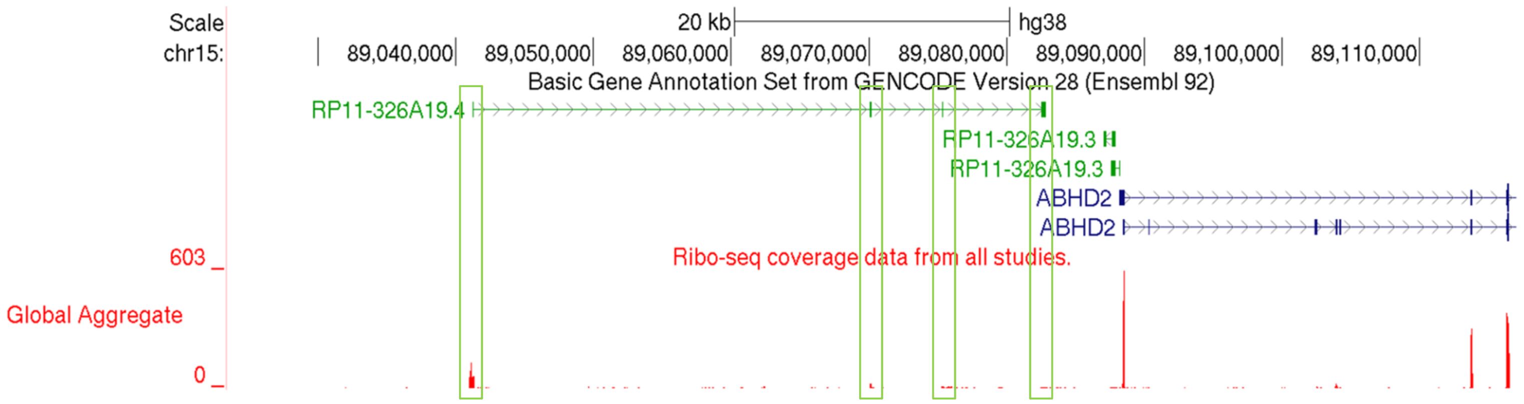

MPCGTEMNCRCRALPTLQNHEQICCGLAVTLLWDPG
MWNRDELPLPSSAHVAES
MEWMIEWMNELHRKMRKAFMDGVSLQQSLKGPTWNAMLPHRQCCPTKFSP
MLWIGCNSALGSRLKEQALSGTLLVSRQKKKRQWNG
MGLHDSVFLVLDVLLSKKCLLSCKLI
MNCRCRALPTLQNHEQICCGLAVTLLWDPG
MLPYQIQPIDLQKPIQISKNPSQKKLLPPLNNYC
MIEWMNELHRKMRKAFMDGVSLQQSLKGPTWNAMLPHRQCCPTKFSP

Figure S8. Ribosome occupancy of CARMAT and possible ORF. Top, aggregate of 9 distinct studies assessing ribosomal footprints in HEK293T. As a quality control, only studies showing *ABHD2* signals in exon 1 were included (9 out of 11). Data obtained via GWIPS-viz (https://gwips.ucc.ie). Bottom, peptides compatible with the ORF. Only ORF longer than 10 aa starting with a Met residue were considered. All 3 frames were considered, throughout the entire transcript.
