# Supplementary material for: CARMAL Is a Long Non-coding RNA Locus That Regulates MFGE8 Expression
Source: Front Genet. 2020 Jun 17;11:631. doi: 10.3389/fgene.2020.00631 (PMC7311772; doi:10.3389/fgene.2020.00631)
Supplement: Supplementary file 3 [file Data_Sheet_3.docx]

**qPCR oligos (Forward/Reverse)**

**ABHD2**

TGAGGTCGCCACATCCTTAT

GCAGATGACCATGGTGATATC

**HAPLN3**

GCAAAGAGCTGTCCCACCTT

CCTGCACCGCTATGATGTATTC

**MFGE8**

CGGTGGTTTATGCGAGG

TGTCCAGGCATTGACCATG

**PPIA**

ACCGTGTTCTTCGACATTGC

TTCTGTGAAAGCAGGAACCC

**RP11-326A19/CARMAL**

Exon 1-2 (default pair)

GCTGATGCCATGTGGAACAG

CTTCTGCCTGGAGACTAGTAATG

Exon 2-3:

CTGGAACATTACTAGTCTCCAGG

TCTGCTGCAAGCTCACTC

Exon 3-4:

GGATGATTGAATGGATGAATGAACTCC

GAGATAACTCAAAGGACTGAGC

**SRP14**

ACTTCCGGCTCTCACTGCTA

TCAAAGCCCTCCACAGTACC

**U1**

atacttacctggcaggggag

CAGGGGAAAGCGCGAACGCA

**Cloning oligos**

All cloned in Bbs1 site of pCRU6

***Single guide RNA oligonucleotides; cloning primer sequences***

CRISPRa oligos (targeted to upstream region of *CARMAL*):

caccAGCTGTTCGGGGATAGCTG

aaacCAGCTATCCCCGAACAGCT

caccTTCAGACAGACAGACACTT

aaacAAGTGTCTGTCTGTCTGAA

caccGGGAGGAGACTACACACAG

aaacCTGTGTGTAGTCTCCTCCC

CRISPRi oligos (targeted to intron 1 of *CARMAL*):

caccGGTGGTTTGCAATAGAATGG

aaacCCATTCTATTGCAAACCACC

caccGGCGGGTGAATACTGATGGG

aaacCCCATCAGTATTCACCCGCC

caccGTAGATCCAGACATCAAGGA

aaacTCCTTGATGTCTGGATCTAC

CRISPR oligos (targeted to upstream and intron 1 regions of *CARMAL*):

caccGGTGGTTTGCAATAGAATGG

aaacCCATTCTATTGCAAACCACC

caccGGGAGGAGACTACACACAG

aaacCTGTGTGTAGTCTCCTCCC

**Antibodies**

SBDS (N1C3, GeneTex)

TUBB (D66, GeneTex)
